# Arteriolosclerosis CSVD: a common cause of dementia and stroke and its association with cognitive function and total MRI burden

**DOI:** 10.3389/fnagi.2023.1163349

**Published:** 2023-07-14

**Authors:** Min Hua, Ai-Jin Ma, Zhi-Qing Liu, Li-Li Ji, Jin Zhang, Yuan-Feng Xu, Wen-Ya Chen, Lun-Lin Mao

**Affiliations:** Department of Neurology, Wujin Hospital Affiliated to Jiangsu University, The Wujin Clinical College of Xuzhou Medical University, Changzhou, Jiangsu, China

**Keywords:** cerebral small vessel disease, white matter hyperintensities, lacunar infarcts, cerebral microbleed, cerebral atrophy, cognitive impairment

## Abstract

**Objective:**

Arteriolosclerosis cerebral small vessel disease (CSVD) is a common type of CSVD. This study aimed to explore the factors associated with cognitive function and total MRI burden related to the disease.

**Methods:**

The demographic characteristics, clinical manifestations, cognitive function score, Barthel Index (BI), blood test index, and follow-up results of arteriolosclerosis CSVD patients treated for the first time in our hospital from January 2014 to August 2022 were collected. White matter hyperintensity (WMH) Fazekas score, total MRI burden, and cerebral atrophy grade were evaluated according to brain MRI findings. Factors associated with CSVD cognitive function were analyzed by binary logistic regression. The correlative factors related to the total MRI burden of CSVD were analyzed by ordered multiple logistic regression.

**Results:**

A total of 146 patients were included in this study, of which 132 cases (90.4%) had hypertension. There were 108 patients (74.0%) with cognitive dysfunction, 97 patients (66.4%) with balance and gait disorders, and 83 patients (56.8%) with moderate-to-severe dependence in daily life (BI ≤ 60 points). Of 146 patients, 79 (54.1%) completed clinical and imaging follow-ups for a median of 3 years. The number of patients with cognitive impairment and BI ≤ 60 points after follow-up significantly increased compared with the first admission (*P* < 0.001). There were also significant differences in total MRI burden (*P* = 0.001), WMH Fazekas score, and cerebral atrophy grade (*P* < 0.001). Mean age (*P* = 0.012), median deep WMH Fazekas score (*P* = 0.028), and median deep (*P* < 0.001) and superficial (*P* =0.002) cerebral atrophy grade of patients with cognitive impairment at first admission were all higher than those with non-cognitive impairment. Multivariate analysis showed that deep cerebral atrophy was independently and significantly associated with cognitive impairment of CSVD (*P* = 0.024), and hypertension was significantly and independently associated with total MRI burden (*P* = 0.001).

**Conclusion:**

The disease course of arteriolosclerosis CSVD may be related to cognitive function and total MRI burden. Deep cerebral atrophy was an independent risk factor for cognitive dysfunction in arteriolosclerosis CSVD, and hypertension was an independent risk factor for total MRI burden.

## Introduction

Cerebral small-vessel disease (CSVD) refers to a series of clinical, imaging, and pathological syndromes caused by various etiologies affecting cerebral arterioles and their distal branches, arterioles, capillaries, capillary vein, and venules. The common etiological types of CSVD include arteriolosclerosis, sporadic or inherited cerebral amyloid angiopathy (CAA), other inherited cerebral small vascular lesions, inflammation, immune-mediated small vascular disease, venous collagen disease, and other small vascular diseases (Pantoni, 2010). The two most common pathologies underlying CSVD are arteriolosclerosis caused by aging, hypertension, and other conventional vascular risk factors, and CAA caused by vascular deposition of β-amyloid.

CSVD is a common cause of dementia and stroke. According to stroke classification, CSVD caused by small artery occlusion accounts for about 30% of the causes of ischemic stroke in China (Wu et al., 2019). The clinical manifestations of CSVD are heterogeneous and can be categorized into acute ischemic CSVD and chronic clinical syndrome with occult onset. Acute ischemic CSVD presents as a specific lacunar syndrome, while chronic CSVD may be asymptomatic and can be diagnosed by imaging. As the burden of CSVD increases gradually, patients may develop symptoms, such as mild cognitive dysfunction, dementia, mood disorders, motor and gait dysfunction, and urinary incontinence. CSVD is diagnosed on the basis of brain imaging biomarkers, including recent small subcortical infarct (RSSI), also known as lacunar infarct, lacune of vascular origin, white matter hyperintensity (WMH) of vascular origin, perivascular space (PVS), cerebral microbleed (CMB), and cerebral atrophy (Wardlaw et al., 2013b). Other imaging features include intracerebral hemorrhage due to single perforator artery lesions, iron deposition on the cortical surface, and cortical microinfarction. Four closely related features on brain MRI, including lacunar, WMH, CMB, and enlarged PVS, are markers of CSVD (Pantoni, 2010; Wardlaw et al., 2013a). Cerebral atrophy may also be associated with CSVD (Appelman et al., 2009; Nitkunan et al., 2011; Aribisala et al., 2013). Individual CSVD features are also associated with vascular risk factors (Ovbiagele and Saver, 2006; Vermeer et al., 2007; Greenberg et al., 2009; Doubal et al., 2010).

CSVD usually has both clinical and imaging manifestations, while CSVD imaging markers may not be accompanied by clinical manifestations. Even in the presence of objective clinical features, these radiological features of CSVD are important, being strong predictors of both stroke and dementia risk. Currently, most studies pay more attention to non-acute with cognitive and gait disorders as clinical manifestations and non-acute CSVD imaging markers without obvious clinical manifestations. Few studies have focused solely on arteriolosclerosis CSVD in combination with these features of CSVD. Therefore, we collected clinical information, imaging data, and follow-up results of previously diagnosed CSVD and used the “CSVD imaging total MRI burden score” to evaluate global brain injury (Klarenbeek et al., 2013; Staals et al., 2014) and the predefined visual standards from the Cardiovascular Health Study to assess cerebral atrophy (Manolio et al., 1994; Yue et al., 1997), to analyze risk factors, clinical features, and imaging features of arteriolosclerosis CSVD, and to explore the correlative aspects of cognitive function and total MRI burden in arteriolosclerosis CSVD.

Recent studies have shown that intensive blood pressure lowering to a systolic of 120 mmHg was associated with both reduced WHM progression (SPRINT MIND Investigators for the SPRINT Research Group et al., 2019a) and a reduction in the combined endpoint of mild cognitive impairment and dementia (SPRINT MIND Investigators for the SPRINT Research Group et al., 2019b). However, this study is a single-center retrospective study on the case data of arteriolosclerosis CSVD, and no further analysis was made on the control of conventional vascular risk factors. Therefore, as in other areas of stroke research, large international collaborative ventures are vital to take the field forward, and the need for large-scale prospective randomized controlled trials of arteriolosclerosis CSVD to demonstrate whether control of other risk factors reduces total MRI burden and delays the onset of cognitive impairment.

## Methods

### Subjects

This was a retrospective study. From January 2014 to August 2022, 146 patients with arteriolosclerosis CSVD were first hospitalized in Wujin Hospital affiliated to Jiangsu University, and were included in this study. Inclusion criteria included brain MRI indicating at least one of the following manifestations: (1) RSSI: neuroimaging evidence of recent infarction in the territory of one perforating arteriole, with imaging features or clinical symptoms consistent with a lesion occurring in the previous few weeks, the maximum diameter of lesion < 20 mm in the axial plane. (2) Lacune of vascular origin: a round or ovoid, subcortical, fluid-filled cavity (signal similar to CSF) of between 3 mm and about 15 mm in diameter, consistent with a previous acute small subcortical infarct or hemorrhage in the territory of one perforating arteriole. (3) WMH of vascular origin: signal abnormality of variable size in the white matter that shows hyperintensity on T2-weighted images such as fluid-attenuated inversion recovery, without cavitation (signal different from CSF). Lesions in the subcortical gray matter or brainstem are not included in this category. (4) CMB: small (generally 2–5 mm in diameter, but sometimes up to 10 mm) areas of the signal void with associated blooming seen on T2^*^-weighted MRI or other sequences that are sensitive to susceptibility effects. (5) PVS: fluid-filled spaces that follow the typical course of a vessel as it goes through gray or white matter. The spaces have a signal intensity similar to CSF on all sequences. Because they follow the course of penetrating vessels, they appear linear when imaged parallel to the course of the vessel, and round or ovoid, with a diameter generally smaller than 3 mm, when imaged perpendicular to the course of the vessel. (6) Cerebral atrophy: a lower brain volume that is not related to a specific macroscopic focal injury such as trauma or infarction. Exclusion criteria included the following: (1) symptomatic intracranial or extracranial large artery (diameter > 2 mm) stenosis and occlusions, asymptomatic extracranial carotid artery stenosis ≥ 70%; (2) non-lacunar infarction (infarct lesion with maximum diameter > 20 mm) caused by large vascular lesions (intracranial or extracranial large artery stenosis and occlusions) or cardiogenic embolism, cerebral amyloid vascular disease, aneurysms or arteriovenous malformations, and other cerebral bleeds; (3) special causes of white matter lesions (multiple sclerosis, sarcoidosis, radiotherapy); (4) brain lesions (such as from Alzheimer's disease, dementia with Lewy bodies, frontotemporal dementia, Parkinson's disease, tumors, hydrocephalus, trauma, syphilis, acquired immune deficiency syndrome, and Creutzfeldt–Jakob disease); (5) severe mental illness, epilepsy, alcohol or drug abuse, intoxication, and metabolic abnormalities; and (6) inflammation- or immune-mediated small cerebral vascular disease, venous collagen disease, or hereditary CSVD.

All patients had well-documented medical histories, complete laboratory examinations (routine blood tests and biochemical indicators), and brain MRI, CT angiography, or magnetic resonance angiography examinations. In this study, all personal identifiers were removed or anonymized during data collection and analysis to protect the privacy of the participants. The study was conducted under the Declaration of Helsinki, and all participants provided informed consent.

### General patient characteristics

General characteristics of all patients were collected based on medical records, including age, sex, education, body mass index (BMI), smoking, hypertension, diabetes, hypercholesterolemia, hyperhomocysteinemia, stroke, transient ischemic attack, ischemic heart disease, and history of atrial fibrillation. Hypertension was defined as blood pressure ≥ 140/90 mmHg, diabetes as fasting blood glucose ≥ 7.0 mmol/L, hypercholesterolemia as total cholesterol ≥ 5.18 mmol/L or LDL cholesterol > 3.12 mmol/L, hyperhomocysteinemia as homocysteine (Hcy) > 15 μmol/L, ischemic heart disease as myocardial infarction, angina, or evidence of myocardial ischemia on the electrocardiogram (ECG), atrial fibrillation as previously diagnosed or found on the ECG, and smoking as currently smoking. At the same time, the main clinical manifestations (core symptoms) of all patients were recorded, and laboratory tests were performed within 7 days after admission, including routine blood tests, liver and kidney function, fasting blood glucose, blood lipid, folic acid, vitamin B12, and homocysteine. Cerebrovascular status was assessed by carotid Doppler ultrasound, CTA, or MRA. Asymptomatic extracranial carotid stenosis < 70% of the patients were still enrolled in the study. The Barthel Index (BI) scale was used to evaluate the activities of daily living (ADL), with a score ranging from 0 to 100 and a higher score indicating greater independence (Pistoia et al., 2016). Self-care ability was divided into four levels: severe dependence, moderate dependence, mild dependence, and no dependence. A BI score ≤ 40 indicated severe dependence, 41–60 indicated moderate dependence, 61–99 indicated mild dependence, and 100 indicated no dependence. The Mini-Mental State Examination (MMSE) was used to evaluate cognitive function, and the MMSE points for cognitive impairment screening were 17 for illiterate, 20 for individuals with 1–6 years of education, and 24 for individuals with 7 or more years of education (Li et al., 2016).

### Brain MRI analysis

All patients completed a brain MRI examination (1.5T, Siemens, Germany), including axial diffusion-weighted imaging (DWI) scan, with a pulse repetition time (TR), 3,000 milliseconds; echo time (TE), 79.5 milliseconds; thickness, 5 mm; gap, 1.5 mm; and matrix, 210 × 256; axial T1-weighted imaging (T1WI) scan, with TR, 451 milliseconds; TE, 12.5 milliseconds; thickness, 5 mm; gap, 1.5 mm; and matrix, 214 × 352; axial T2-weighted imaging (T2WI) scan, with TR, 4,500 milliseconds; TE, 82.9 milliseconds; thickness, 5 mm; gap, 1.5 mm; and matrix, 233 × 384; axial fluid-attenuated inversion recovery (FLAIR) scan, with TR, 8,000 milliseconds; TE, 75.9 milliseconds; thickness, 5 mm; gap, 1.5 mm; and matrix, 155 × 256; and axial susceptibility weighted imaging (SWI) scan, with TR, 50.1 milliseconds; TE, 40 milliseconds; thickness, 4 mm; gap, 2 mm; and matrix, 194 × 320. All axial scan planes were parallel to the anterior commissure–posterior commissure (AC/PC) line.

All MRI images were assessed blinded to clinical information by one experienced neuroradiologist and one neurologist for the presence, location, and size of the recent symptomatic infarct and any other vascular lesions. (1) RSSI: the imaging features were round or ovoid lesions located in the area of perforating arteries, with low signal on the T1WI sequence, with a hyperintense area on DWI, T2WI, and FLAIR. The diameter of the lesions was 3–20 mm. The lesions were mainly distributed in the basal ganglia, internal capsule, centrum semiovale, or brainstem. (2) Lacune: a round or ovoid, in the basal ganglia, internal capsule, centrum semiovale, or brainstem, a fluid-filled cavity of between 3 mm and about 15 mm in diameter, with a CSF signal intensity on T1WI, T2WI, and FLAIR, generally with a hyperintense rim on FLAIR and no increased signal on DWI. (3) WMH: in the early stage, it was a small cap lesion in the frontal lobe and the occipital horn. The progression of the lesion may extend to the subcortical white matter area and fuse, showing abnormal signals of varying sizes in the white matter area of the brain. It offers a high signal on T2WI and FLAIR and an equal or low signal on T1WI. (4) CMB: with the paramagnetic properties of hemosiderin-containing blood breakdown products. Small round or ovoid lesion, well-defined, and homogenous signal loss foci were seen on SWI in the cerebellum, brainstem, basal ganglia, white matter, or cortico-subcortical junction. However, it was not visible on FLAIR, T1WI, and T2WI. The diameter ranges from 2 to 5 mm, with a maximum of 10 mm, differentiated from vessel flow voids and mineral depositions in the globi pallidi. (5) PVS: they appeared round in the axial section and linear if in longitudinal sections and were smaller than 3 mm wide. They were of high signal on T2WI and low signal on T1WI and FLAIR sequences. They were distinguished from lacunes by the latter's large size (more than 3 mm and spheroid shape). PVS mainly occurred in the basal ganglia, subcortical, and brain stem, but rarely in the cerebellum. (6) Cerebral atrophy: it was classified as both deep (enlargement of the ventricles) and superficial (enlargement of the sulci), and MRI showed that the brain volume was reduced, the ventricle was enlarged, and the sulci and gyrus were widened.

### Total MRI burden score of CSVD

A score scale proposed by Staals et al. (2014) was used to comprehensively evaluate the total MRI burden of CSVD, including the lacune, WMH, CMB, and PVS, the four most typical CSVD imaging findings. Deep and periventricular WMH were coded according to the Fazekas scale from 0 to 3 (Fazekas et al., 1987). Periventricular WMH scoring was 0 = no lesions; 1 = cap or pencil thin-layer lesions; 2 = the lesion showed a smooth halo; and 3 = irregular periventricular WMH extending into the deep white matter. Deep WMH scoring was 0 = no lesions; 1 = spotty lesions; 2 = the lesions were beginning to fuse; and 3 = large area fusion of lesions. PVS in both the basal ganglia and centrum semiovale were coded with the following scale applied to standard axial images (Doubal et al., 2010): 0 = no PVS; 1 = < 10 PVS; 2 = 11–20 PVS; 3 = 21–40 PVS; and 4 = >40 PVS. The numbers refer to PVS on one side of the brain; the higher score was used if there was an asymmetry between the sides, and PVS was counted in the slice with the highest number. The total MRI burden of CSVD on an ordinal scale from 0 to 4 by measuring the presence of each of the four MRI features of CSVD. A point was awarded for each of the following: the presence of lacunes and CMB, defined as the presence of one or more lacunes (1 point if present) or any CMB (1 point if present). The presence of PVS was counted if there were moderate-to-severe (grade 2–4) PVS in the basal ganglia (1 point if present). The presence of WMH was defined as either (early) confluent deep WMH (Fazekas score 2 or 3) or irregular periventricular WMH extending into the deep white matter (Fazekas score 3, 1 point if present).

### Cerebral atrophy assessment

Cerebral atrophy is characterized by reduced brain volume and is not associated with local volume reduction caused by brain trauma and cerebral infarction. Cerebral atrophy was classified as either deep (enlargement of the ventricles) or superficial (enlargement of the sulci). Reduced brain volume, enlarged ventricles, and widened sulci were seen on brain MRI. The sulcal prominence and ventricular size in each individual were assessed on a semiquantitative 10-point scale (grades of 0–9) using predefined visual standards from the Cardiovascular Health Study (Manolio et al., 1994; Yue et al., 1997). According to the T1-weighted images for grading ventricular and sulcal size, the atrophy grade is determined by visual comparison with templates indicating normal to atrophied brains obtained in Cardiovascular Health Study, sulcal widening and a ventricular size ranging from small and presumably normal (grade 1) to severe enlargement (grade 8). Studies considered having sulci or ventricles smaller than those in grade 1 received a grade of 0, and those worse than grade 8 received a grade of 9.

### Statistical analysis

Continuous variables were described as means ± standard deviations or medians (interquartile ranges, IQR) based on whether data were normally distributed or not. Categorical variables were presented as frequencies and percentages. Two independent sample *t*-test, the Wilcoxon rank sum test and the chi-square test, were used to compare the differences between patients with cognitive impairment and non-cognitive impairment at the first admission. Paired-samples *t*-test, Wilcoxon rank sum test and chi-square test were used to compare the differences in clinical and imaging characteristics between the first admission and follow-up. The binary logistic regression analysis was subsequently performed to identify the independent risk factors for CSVD cognitive impairment, of which those variables with *P* < 0.05 in the univariate analysis and clinical parameters that may be closely related to CSVD cognitive impairment in light of clinical experience and previous research were included, such as age, sex, history of TIA or stroke, WMH, total MRI burden, cerebral atrophy, and risk factors that are frequently associated with individual features of arteriolosclerosis CSVD, i.e., hypertension, diabetes mellitus, and hyperhomocysteinemia. Finally, we performed univariate and multivariable ordinal logistic regression analyses related to the total MRI burden with age, sex, smoking, history of TIA or stroke, cerebral atrophy, hypertension, diabetes mellitus, hyperhomocysteinemia, and hypercholesterolemia, and the results were expressed as odds ratio (OR) and 95% confidence interval (CI). Analyses were performed with SPSS version 21.0, and statistical significance was set at *P* < 0.05.

## Results

### Patients' characteristics

A total of 146 patients with arteriolosclerosis CSVD were enrolled in this study from January 2014 to August 2022. The mean age of the patients was 75.13 ± 8.93 years old (age range: 46–100 years old), 88 cases (60.3%) were male, 132 cases (90.4%) were complicated with hypertension, 40 patients (27.4%) presented with diabetes mellitus, 27 cases (18.5%) presented with hypercholesterolemia, 71 cases (48.6%) presented with hyperhomocysteinemia, and 57 patients (39.0%) were overweight. There were 17 cases (11.6%) with asymptomatic extracranial carotid artery stenosis on the first admission. There were 123 cases (84.2%) of transient ischemic attack (TIA) or stroke in previous or first diagnosis (Table 1), including 100 cases (68.5%) of TIA or cerebral infarction, eight cases (5.5%) of cerebral hemorrhage, and 15 cases (10.3%) of cerebral infarction and cerebral hemorrhage. The median NIHSS score for patients with cerebral infarction admitted for the first time was 3 (IQR 2–4).

**Table 1:** Clinical and imaging characteristics of all patients at first admission.

**Characteristics**	**All patients (*n* = 146)**	**Cognitive impairment patients (*n* = 108)**	**Non-cognitive impairment patients (*n* = 38)**	***P***
Age, y	75.13 ± 8.93	76.22 ± 8.32	72.03 ± 9.95	0.012
Male sex	88 (60.3)	64 (59.3)	24 (63.2)	0.673
BMI (overweight)	57 (39.0)	42 (38.9)	15 (39.5)	0.949
BI ≤ 60 points	83 (56.8)	73 (67.6)	10 (26.3)	0.000
Ischemic heart disease	6 (4.1)	6 (5.6)	0 (0.0)	0.313
Diabetes mellitus	40 (27.4)	27 (25.0)	13 (34.2)	0.274
Hypertension	132 (90.4)	98 (90.7)	34 (89.5)	1.000
Atrial fibrillation	7 (4.8)	7 (6.5)	0 (0.0)	0.243
Smoking	32 (21.9)	23 (21.3)	9 (23.7)	0.760
Hyperhomocysteinemia	71 (48.6)	54 (50.0)	17 (44.7)	0.577
Hypercholesterolemia	27 (18.5)	22 (20.4)	5 (13.2)	0.325
Hyperuricemia	27 (18.5)	18 (16.7)	9 (23.7)	0.338
Chronic renal insufficiency	27 (18.5)	22 (20.4)	5 (13.2)	0.325
Folate deficiency	21 (14.4)	18 (16.7)	3 (7.9)	0.185
Vitamin B12 deficiency	19 (13.0)	16 (14.8)	3 (7.9)	0.418
Asymptomatic extracranial carotid artery stenosis (< 70%)	17 (11.6)	12 (11.1)	5 (13.2)	0.965
Anemia	19 (13.0)	17 (15.7)	2 (5.3)	0.170
History of TIA or stroke	123 (84.2)	91 (84.3)	32 (84.2)	0.994
**MRI feature**				
Lacune	144 (98.6)	108 (100.0)	36 (94.7)	0.066
CMB	136 (93.2)	100 (92.6)	36 (94.7)	0.939
PVS (grade 2–4)	73 (50.0)	53 (49.1)	20 (52.6)	0.706
Periventricular WMH Fazekas score	3 (3–3)	3 (3–3)	3 (2–3)	0.150
Deep WMH Fazekas score	2 (1–2)	2 (1–2)	1 (1–2)	0.028
Total MRI burden score	3 (3–4)	3 (3–4)	3 (3–4)	0.226
Superficial cerebral atrophy grade	4 (3–5)	4 (3–5)	2 (2–4)	0.002
Deep cerebral atrophy grade	5 (4–6)	5 (4–6)	4 (3–5)	0.000

BMI, body mass index; BI, Barthel index; hsCRP, high-sensitivity C-reactive protein; TIA, transient ischemic attack; MRI, magnetic resonance imaging; CMB, cerebral microbleed; PVS, perivascular space; WMH, white matter hyperintensity. Data presented as mean ± SD or number (%) or median (interquartile range).

### Clinical manifestation

At the first admission of all patients, 97 patients (66.4%) had balance and gait disorders, 69 patients (47.3%) had limb weakness, 65 patients (44.5%) had speech disorders, and 25 patients (17.1%) had dizziness or headache. Additionally, 12 patients (8.2%) had emotional and behavioral abnormalities, and 11 patients (7.5%) had dysphagia. According to the ADL assessment, 83 patients (56.8%) had moderate-to-severe dependence in daily life (BI ≤ 60). MMSE assessment indicated that 108 patients (74.0%) had cognitive impairment (Table 2).

**Table 2:** Clinical manifestation of all patients at first admission.

**Characteristics**	**All patients (*n* = 146)**
**Core symptoms**	
Cognition impairment	108 (74.0)
Balance and gait disorder	97 (66.4)
Limb weakness	69 (47.3)
Speech disorder	65 (44.5)
Dizziness or headache	25 (17.1)
Emotional and behavioral abnormalities	12 (8.2)
Dysphagia	11 (7.5)
Sensory disorder	7 (4.8)
Disturbance of consciousness or seizures	5 (3.4)
Bowel and urination disorders	4 (2.7)
Visual impairment	2 (1.4)
**BI**	
100 points	14 (9.6)
61–69 points	49 (33.6)
41–60 points	42 (28.8)
≤ 40 points	41 (28.1)

BI, Barthel index.

### Brain MRI manifestations and total MRI burden

A brain MRI analysis of all patients at the first admission revealed that 73 cases (50.0%) had RSSI, six cases (4.1%) had a cerebral hemorrhage, 144 cases (98.6%) had lacune, and 136 cases (93.2%) had CMB. Meanwhile, 73 patients (50.0%) had PVS (grade 2–4), 145 cases (99.3%) had WMH, the total WMH Fazekas score median was 5 (IQR 4–5), and the periventricular WMH Fazekas score median was 3 (IQR 3–3). The median deep WMH Fazekas score was 2 (IQR 1–2). The median score of total MRI burden was 3 (IQR 3–4) points, including 53 cases (36.3%) with four points, 80 patients (54.8%) with three points, and 13 cases with two points (8.9%). Furthermore, 146 cases (100.0%) all had different degrees of cerebral atrophy according to the visual standards from the Cardiovascular Health Study, with a median superficial cerebral atrophy grade of 4 (IQR 3–5) and a median deep cerebral atrophy grade of 5 (IQR 4–6) (Table 1).

### Comparison of clinical and imaging characteristics of patients at first admission and follow-up

**Figure 1:**
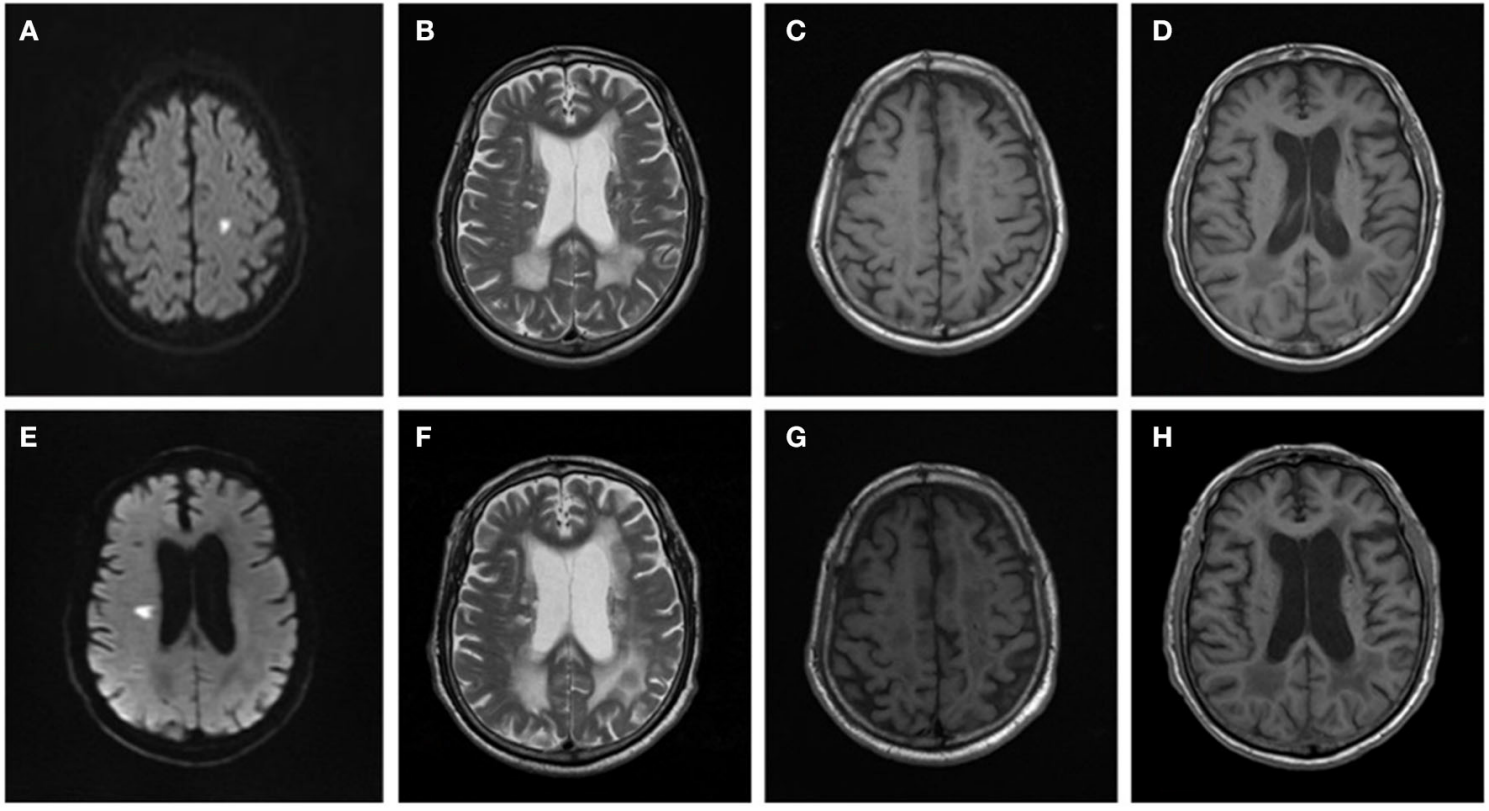
Brain MRI changes in a patient with arteriolosclerosis CSVD. Male, 66 years old, was admitted to the hospital due to right limb weakness for 2 days in 2014. Brain MRI: **(A)** DWI showed a small flake of high signal in the left centrum semiovale. **(B)** T2WI showed irregular hyperintensity near the ventricle. **(C, D)** T1WI showed enlargement of the sulci and slight enlargement of the ventricles. In 2018, the patient was readmitted to the hospital due to dysarthria and left limb weakness for 1 day. Brain MRI: **(E)** DWI showed a small flake of high signal near the right lateral ventricle. **(F)** T2WI, the range of irregular hyperintensity near the ventricle was enlarged and extended to deep white matter with large area fusion. **(G, H)** T1WI showed enlargement of the sulci and enlargement of the ventricles, which made progress compared with 2014.

There were 79 (54.1%) patients who completed clinical and imaging follow-up for more than 6 months, of which 75 patients had hypertension. The median clinical and imaging follow-up was 3 (IQR 2–5) years, with 48 patients followed up for more than 3 years and 28 patients followed up for more than 5 years. During the follow-up period, 32 cases (32/79, 40.5%) had an average of one to three TIA or stroke events, of which one case had TIA, 29 patients had a new small infarction with a median NIHSS score of 4 (IQR 2–4), and two cases had a cerebral hemorrhage. After a median follow-up of 3 years, the average age of patients increased significantly and the number of patients with cognitive impairment was significantly higher than at the time of initial admission (70/79 vs. 54/79, *P* < 0.001). There was also a significant increase in the number of people with moderate-to-severe dependence on daily living requirements compared with the first admission (64/79 vs. 41/79, *P* < 0.001). During follow-up, the WMH range and the degree of cerebral atrophy progressed with the prolongation of the disease course (Figure 1). At follow-up, although the periventricular and deep WMH Fazekas scores and median total MRI burden scores were the same as those at first admission, the overall distribution of the total MRI burden (*P* = 0.001) and the WMH Fazekas score (*P* < 0.001) were significantly different. The median of deep and superficial cerebral atrophy grades was significantly higher than that at the first admission (*P* < 0.001) (Table 3).

**Table 3:** Comparison of clinical and imaging characteristics of patients at the first admission and follow-up.

**Characteristics**	**First admission patients (*n* = 79)**	**Follow-up patients (*n* = 79)**	***P***
Age	71.75 ± 8.94	75.55 ± 9.86	0.000
Cognition impairment	54 (68.4)	70 (88.6)	0.000
BI ≤ 60 points	41 (51.9)	64 (81.0)	0.000
Lacune	79 (100.0)	79 (100.0)	–
WMH	79 (100.0)	79 (100.0)	–
CMB	75 (94.9)	75 (94.9)	1.000
PVS (grade 2–4)	38 (48.1)	39 (49.4)	1.000
Periventricular WMH Fazekas score	3 (2–3)	3 (3–3)	0.000
Deep WMH Fazekas score	2 (1–2)	2 (2–3)	0.000
Total MRI burden score	3 (3–4)	3 (3–4)	0.001
4 points	25 (31.6)	34 (43.0)	0.004
≤ 3 points	54 (68.4)	45 (57.0)	
Superficial cerebral atrophy grade	3 (3–4)	5 (4–5)	0.000
Deep cerebral atrophy grade	5 (4–6)	6 (5–7)	0.000

BI, Barthel index; CMB, cerebral microbleed; WMH, white matter hyperintensity; PVS, perivascular space; MRI, magnetic resonance imaging.

Data presented as mean ± SD or number (%) or median (interquartile range).

### Comparison of clinical characteristics between patients with cognitive impairment and non-cognitive impairment at the first admission

The mean age of the patients with cognitive impairment at first admission was 76.22 ± 8.32 years, which was higher than those with non-cognitive impairment (72.03 ± 9.95 years, *P* = 0.012). The proportion of severely dependent patients (BI ≤ 60) with cognitive impairment was significantly higher than those with non-cognitive impairment (*P* < 0.001). There were no significant differences in total MRI burden scores between the two groups (*P* = 0.226), but the median deep WMH Fazekas score (*P* = 0.028), deep (*P* < 0.001), and superficial (*P* = 0.002) cerebral atrophy grades of patients with cognitive impairment at first admission were all higher than those with non-cognitive impairment. There were no significant differences in other clinical characteristics (Table 1). In multivariate analysis, deep cerebral atrophy was independently and significantly associated with cognitive impairment after correction for important confounders, including vascular risk factors, age, WMH, and total MRI burden (*P* = 0.024) (Table 4).

**Table 4:** Multivariate analysis of cognitive impairment associated with CSVD.

**Factors**	***P***	**OR (95% CI)**
Age	0.735	1.175 (0.462–2.983)
Male sex	0.416	1.022 (0.969–1.078)
Hypertension	0.588	1.532 (0.328–7.166)
Diabetes mellitus	0.251	0.599 (0.250–1.435)
Hyperhomocysteinemia	0.870	1.073 (0.462–2.494)
Periventricular WMH	0.581	0.757 (0.281–2.037)
Deep WMH	0.271	1.437 (0.754–2.738)
Total MRI burden	0.484	1.303 (0.621–2.730)
Superficial cerebral atrophy	0.068	1.481 (0.971–2.260)
Deep cerebral atrophy	0.024	1.491 (1.054–2.109)
History of TIA or stroke	0.557	0.707 (0.223–2.245)

CSVD, cerebral small vessel disease; CI, confidence interval; OR, odds ratio; WMH, white matter hyperintensity; MRI, magnetic resonance imaging; TIA, transient ischemic attack.

Multivariable analysis with correction for all variables in the table.

### Univariate and multivariate analyses of the total MRI burden

In univariate analysis, the total MRI burden of CSVD was associated with smoking (OR 2.192, 95% CI 1.010–4.754, *P* = 0.047) and hypertension (OR 4.904, 95% CI 1.547–15.549, *P* = 0.007). However, there was no significant association with sex, age, diabetes, hypercholesterolemia, hyperhomocysteinemia, TIA or stroke history, or cerebral atrophy. In multivariable analysis, after correction for important confounders, including vascular risk factors, age, smoking, and cerebral atrophy, hypertension was significantly and independently associated with total MRI burden in arteriolosclerosis CSVD (OR 7.338, 95% CI 2.175–24.730, *P* = 0.001) and the association for hypertension becoming stronger in multivariable than in univariate testing (Table 5).

**Table 5:** Univariate and multivariate analysis of the total MRI burden.

**Factors**	**Univariate**		**Multivariate**	
	***P***	**OR (95% CI)**	***P***	**OR (95% CI)**
Male sex	0.058	1.898(0.979–3.677)	0.119	1.921(0.845–4.371)
Age	0.724	1.006(0.971–1.043)	0.481	1.016(0.971–1.063)
Smoking	0.047	2.192(1.010–4.754)	0.160	1.923(0.773–4.787)
Hypertension	0.007	4.904(1.547–15.549)	0.001	7.338(2.175–24.730)
Diabetes mellitus	0.891	0.951(0.467–1.937)	0.735	0.880(0.418–1.850)
Hypercholesterolemia	0.933	0.966 (0.427–2.184)	0.778	1.145(0.447–2.930)
Hyperhomocysteinemia	0.354	1.351(0.715–2.552)	0.555	1.230(0.618–2.447)
History of TIA or stroke	0.995	1.003(0.420–2.392)	0.476	0.705(0.270–1.842)
Superficial cerebral atrophy	0.194	1.202(0.911–1.584)	0.208	1.220(0.895–1.664)
Deep cerebral atrophy	0.177	1.165(0.933–1.456)	0.397	1.116(0.865–1.441)

CSVD, cerebral small vessel disease; CI, confidence interval; OR, odds ratio; TIA, transient ischemic attack.

Multivariable analysis with correction for all variables in the table.

## Discussion

Small cerebral vascular disease is an age-related disease, with its prevalence increasing with age. It affects about 5% of people aged 50 to almost 100% of people over 90 years old. Additionally, 25% of strokes and 45% of dementia are caused by CSVD (Cannistraro et al., 2019). Arteriolosclerosis CSVD is the most common type of CSVD. It is well-known that hypertension is a major risk factor for CSVD (Veglio et al., 2009; Meissner, 2016). This study summarized the clinical characteristics of patients with arteriolosclerosis CSVD, whose average age was over 75 years old, and found that hypertension was the main intervention risk factor. In addition, 84.2% of patients had a history of TIA or stroke, mainly in the form of subcortical infarction. This suggests a higher risk of TIA or stroke events in arteriolosclerosis CSVD. Most patients with CSVD have occult onset and various clinical manifestations, including asymptomatic CSVD, lacunar syndrome, and cognitive impairment. The presence and severity of the patient's symptoms depend on the location, extent, and number of lesions. In this study, cognitive impairment was the primary clinical manifestation of CSVD, followed by balance and gait disorder, and with the prolongation of the disease, patients' ADL gradually decreased. Studies have shown that CSVD cognitive dysfunction can present a characteristic pattern of cognitive decline; that is, it has a characteristic early involvement in the areas of attention, processing speed, and executive function, and memory function is relatively intact, which can progress to mild cognitive impairment and vascular dementia (Sachdev et al., 2014). Therefore, attention should be paid to patients with cognitive and gait disorders in clinical practice to identify CSVD as early as possible.

The recurrence rate of stroke caused by CSVD is slightly lower than that caused by atherosclerosis of large vessels. The 1-year recurrence rate of stroke caused by CSVD with hypertension is 14%, while the recurrence rate of stroke caused by CSVD without hypertension is 9.3% (Wang et al., 2013). A total of 79 patients in this study completed clinical and imaging follow-up for more than 6 months, of which 75 patients had hypertension. The median clinical and imaging follow-up was 3 (IQR 2–5) years, and during the follow-up period, 32 cases (40.5%) had an average of one to three TIA or stroke events, which also indicated that arteriolosclerosis CSVD with hypertension had a high risk of stroke. In addition, as the disease course progressed, the incidence of cognitive impairment and reduced ability to schedule life also increased gradually. The total MRI burden, WMH Fazekas score, and cerebral atrophy grade also showed significant changes. Therefore, we speculate that the course of arteriolosclerosis CSVD may be related to cognitive function and total MRI burden.

In this study, the mean age, the deep WMH Fazekas score, and the deep and superficial cerebral atrophy grades of patients with cognitive dysfunction were significantly higher than those without cognitive dysfunction. At the same time, the degree of life dependence of patients with cognitive dysfunction was significantly higher than that of patients with non-cognitive dysfunction. After adjusting for age and other factors, deep cerebral atrophy was independently associated with cognitive dysfunction. Several different grading scales are used for the assessment of cerebral atrophy, including medial temporal atrophy (Scheltens et al., 1992), posterior cerebral atrophy (Koedam et al., 2011), and global cortical atrophy (Pasquier et al., 1996). Because arteriolosclerosis CSVD affects the entire brain, not just part of it, the CHS scale was used in this study to assess the entire superficial cortex and deep cerebral atrophy. While many studies have reported cerebral atrophy associated with CSVD, it is not specific to CSVD and occurs in many other conditions, including normal aging. However, several studies have suggested that cerebral atrophy is a marker of CSVD and modulates the effect of vascular disease on cognition (Levy-Cooperman et al., 2008; Carmichael et al., 2010; Wardlaw et al., 2013b). Other studies reported cerebral atrophy caused by CSVD is a predictor of cognitive dysfunction in CSVD patients (Viswanathan et al., 2010). This study also indicated that cerebral atrophy was closely related to cognitive dysfunction in CSVD patients. However, a visual analog scale was used to evaluate cerebral atrophy, and quantitative brain volume measurements may be required to explore this issue further.

For CSVD, the diagnostic specificity of any single imaging marker is low, and the high MRI burden score may better reflect the overall effect of CSVD on the brain than considering one or two features alone. For example, WMH usually occurs in white matter, while lacune occurs in deep gray matter. Therefore, the total MRI burden score was also used in this study to improve the specificity of CSVD diagnosis. Total MRI burden score was positively correlated with the severity of CSVD, and a study has shown that a higher total MRI CSVD burden was associated with overall cognitive impairment. It was shown that the total MRI CSVD burden score was negatively correlated with the overall MMSE and Montreal Cognitive Assessment Scale (Li et al., 2021). However, there was also a study indicating that no association was found between total MRI burden score and cognitive declines (Hu et al., 2022). Similarly, in this study, there was no significant difference in the total MRI CSVD burden score between the cognitive impairment group and the non-cognitive impairment group. Maybe it will also require a larger sample size research to further explore the correlation between total MRI burden and cognitive impairment.

When analyzing factors associated with total MRI burden in arteriolosclerosis CSVD, hypertension was determined to be an independent risk factor. Yang et al. (2018) found that 24-h day and night systolic blood pressure (SBP) levels and SBP variability were positively related to CSVD burden. Higher SBP levels and SBP variability were independent risk factors for CSVD. Recent studies have also shown that blood pressure variability was associated with a high MRI burden of CSVD imaging markers, especially WMH (Ma et al., 2020). However, the underlying pathophysiological mechanisms between blood pressure levels and the MRI burden of CSVD are complicated and still not fully understood. Studies have confirmed that increased permeability of small vascular walls and the blood–brain barrier contributes to the development of CSVD, and reports have associated CSVD with microvascular endothelial cells and tight junction damage (Pantoni, 2010; Wardlaw et al., 2013a). Higher blood pressure and ambulate blood pressure variability (ABPV) can lead to increased mechanical stress on the vessel wall, resulting in endothelial damage and atherosclerosis (Schillaci et al., 2012; Diaz et al., 2013). Therefore, it has been suggested that higher blood pressure and ABPV promote the development of CSVD through endothelial cell damage (Yang et al., 2018). This study also found that hypertension was independent of the total MRI burden of CSVD, but failed to explore the influence of blood pressure and ABPV on CSVD, which still needs to be investigated by prospective clinical studies and animal experiments with a larger sample size.

In this study, multivariate analysis showed that the effect of hypertension on the total MRI burden was greater than that in univariate analysis, which may be related to the inclusion of variables with protective factors, thereby increasing the OR value of hypertension. Therefore, there are some limitations to our study. First, our study subjects were chosen from hospitalized patients at a single center; thus, cases may not be representative of the general population. Second, this was a retrospective study with limited data and small sample size, which is prone to selection bias and recall bias. So a randomized controlled prospective study with larger samples is needed to explore further the factors affecting cognitive function and total MRI burden of arteriolosclerosis CSVD and to determine whether there is a causal relationship between cognitive function and deep cerebral atrophy, hypertension, and total MRI burden in arteriolosclerosis CSVD patients.

Currently, CSVD has an enormous global impact; uncertainties regarding pathogenesis have delayed the development of effective treatment. The most widely accepted approach to treatment is to intensively control well-established vascular risk factors, of which hypertension is the most important, and intensive blood pressure lowering reduces WMH progression (SPRINT MIND Investigators for the SPRINT Research Group et al., 2019a) and delays the onset of cognitive impairment (SPRINT MIND Investigators for the SPRINT Research Group et al., 2019b), but these studies did not evaluate the efficacy of intensive antihypertensive therapy on RSSI, CMB, or PVS. Therefore, further research is needed to explore the preventive and therapeutic effects of intensive antihypertensive therapy on CSVD in future. Our study will provide important information on the progression of arteriolosclerosis CSVD to cognitive impairment and factors that influence cognitive function and the total MRI burden, and this is a piece of valuable clinical information for clinicians. However, large-scale studies to explore the pathophysiological mechanisms of arteriolosclerosis CSVD and develop novel therapeutic approaches are essential if we take the field forward. With a better understanding of pathogenesis, specific therapies may emerge.

## Conclusion

This study analyzed the clinical features and imaging data of 146 patients with arteriolosclerosis CSVD. Based on our results, it can be speculated that the disease course of arteriolosclerosis CSVD may be related to cognitive function and total MRI burden. Deep cerebral atrophy was an independent risk factor for cognitive impairment in CSVD, and hypertension was an independent risk factor for total MRI burden. Therefore, in clinical practice, we should pay attention to the characteristic MRI features of CSVD, and early identification of brain MRI imaging characteristics of CSVD will provide an opportunity to forestall progression before the emergence of symptoms. Attention should be paid to the common clinical manifestations of CSVD, such as lacunar syndrome and cognitive impairment. Pay attention to the follow-up of arteriolosclerosis CSVD, screening, and intervention of vascular risk factors. Attention should be paid to the evaluation of cognitive function in patients with deep cerebral atrophy and take individualized secondary prevention of stroke for arteriolosclerosis CSVD with hypertension, which may help to delay the progression of arteriolosclerosis CSVD and reduce the risk of disability and dementia in CSVD patients.

## Data availability statement

The original contributions presented in the study are included in the article/supplementary material, further inquiries can be directed to the corresponding authors.

## Ethics statement

The studies involving human participants were reviewed and approved by the Institutional Ethics Committee at Wujin Hospital, Affiliated with Jiangsu University. The patients/participants provided their written informed consent to participate in this study. Written informed consent was obtained from the individual(s) for the publication of any potentially identifiable images or data included in this article.

## Author contributions

L-LM and W-YC were responsible for the conception and design of the study. MH, A-JM, Z-QL, L-LJ, JZ, and Y-FX collected the clinical data, discussed the results, and contributed to the final version of the manuscript. All authors read and approved the final manuscript, contributed toward data analysis, drafting, revising the manuscript, and agreed to be accountable for all aspects of the study.
